# We need less animal research, better animal research, and animal research done for the right reasons

**DOI:** 10.1186/s12967-026-08259-y

**Published:** 2026-05-16

**Authors:** Benjamin Victor Ineichen

**Affiliations:** 1 Center for Reproducible Science and Research Synthesis, University of Zurich, Zurich, Switzerland; 2 Department of Clinical Research, University of Bern, Bern, Switzerland

## Abstract

Animal research has contributed to modern medicine and continues to play an important role in biomedical science. At the same time, its scale and quality deserve closer scrutiny. If it is to retain scientific and societal legitimacy, we must be clear about when we use it, how we conduct it, and why we rely on it at all.

## Clinical trial number

Not applicable.

## Main

Animal research is a cornerstone of biomedical science and has contributed to modern medicine. The discovery of insulin is one clear example: In the early 1920s, experiments in dogs showed that pancreatic extracts could lower blood glucose. Within a year, insulin was used to treat patients with diabetes, turning a fatal disease into a manageable one [1]. Another example is the development of monoclonal antibodies: The hybridoma technique, first established in mice in the 1970s, enabled the production of highly specific antibodies that are now central to the treatment of cancer, autoimmune, and inflammatory diseases [2]. Vaccines are another success, with animal research contributing to their development and safety assessment [3]. These examples show what animal research can achieve. At the same time, past success does not exempt current practice from scrutiny. At a time when biomedical research faces growing concerns about reproducibility, translational failure, and public trust, the way we use animal models has become an urgent scientific and societal question [4, 5]. If animal research is to remain credible, we must examine how and why we use it.

*We need less research, better research, and research done for the right reasons*.

The famous quote by Doug Altman, although originally referring to clinical research [6], equally applies to animal research. The volume of biomedical publications has grown sharply over the past decades, with several hundred thousand animal studies published each year [7]. Yet growth in quantity does not guarantee growth in knowledge. Incentives in academia reward publication counts, novelty, and positive findings over careful design, replication, and critical appraisal of whether a study is needed at all [8]. And decisions to conduct in vivo experiments are not always fully under the control of researchers, as pressure from peer review and publishing, including animal methods bias, can favour their inclusion [9]. In this environment, Altman’s statement is more relevant than ever. The question is not whether animal research is good or bad: The question is whether we are producing work that is necessary, reliable, and aligned with meaningful scientific goals. This is also consistent with the 3Rs (Replacement, Reduction, and Refinement) which remain the main ethical framework for animal use in research [10].

First, we need less animal research. Not less knowledge, but fewer animal studies that are redundant or poorly justified. Too often, new experiments are launched without assessment of what is already known [11]. New animal studies should be grounded in existing evidence, ideally informed by systematic review principles [12]. But even if full systematic reviews might not be feasible due to time constraints [13], methods such as rapid reviews can provide guidance [14]. Similar questions are tested repeatedly, sometimes in slightly modified models, without resolving key uncertainties [15]. Before starting a new animal study, researchers should ask: is this experiment necessary, what is the underlying research question, does it address a meaningful patient-relevant need, and what will it add [16, 17]?

Reduction also means avoiding automatic escalation. Exploratory findings in animal studies should not trigger cascades of follow-up studies unless the signal is robust. Nor should animal experiments be the default when other approaches can answer the same question. New Approach Methodologies (NAMs), including organoids, organ-on-chip systems, and computational models, can address specific mechanistic questions with direct human relevance [18, 19]. Emerging infrastructure can help identify and implement NAMs where appropriate (e.g. [20, 21]). Despite promising advances [22, 23], NAMs also have limitations, including restricted physiological scope and challenges in reporting and reproducibility [24]; initiatives such as the NC3Rs DRIVER guidelines aim to address these issues in part. And in the foreseeable future, they will not fully replace all animal research [25]; although in some contexts they already complement or even supersede it [26]. When a validated non-animal method can provide the answer, repeating the experiment in animals adds little value. Strategic reduction is therefore not anti-science; it is a prerequisite for efficient and responsible science.

Second, we need better animal research. Many studies across neuroscience, oncology, and other fields have documented problems with internal validity (that is, whether the design of a study allows a fair test of the hypothesis without bias). Common shortcomings include lack of randomization (not allocating animals to groups by chance), absence of blinding (researchers knowing which treatment an animal receives), missing sample size calculations, and selective reporting of outcomes [11, 27, 28]. These weaknesses increase the risk of false-positive findings. Practices such as p-hacking (trying multiple analyses until a significant result appears) and HARKing (formulating hypotheses after results are known) further distort the evidence base [29]. When rigor is low, we risk building entire research programs on unstable findings. Improving standards is not about bureaucracy. It is about ensuring that each animal used in research contributes reliable information, a process that can be supported by emerging AI tools for study design, risk-of-bias assessment, and structured reporting [30].

Third, animal research must be done for the right reasons. One recurring problem is low external validity (that is, whether findings from a model generalize to the real-world condition of interest). Animal models are approximations of human disease that capture only parts of the underlying biology. This is not a flaw per se as animal experiments can uncover basic biological mechanisms and drug modes of action [31]. They are particularly valuable in early discovery phases, where understanding pathways and targets is essential. Problems arise when models are used beyond their limits: Testing drug efficacy in animal models that poorly reflect human disease biology often leads to inflated expectations. An example is NXY-059 for acute ischemic stroke. In animal models, including primate studies, it appeared strongly neuroprotective. Yet in large phase III clinical trials (SAINT I and II), it failed to improve outcomes in patients. Subsequent analyses suggested that preclinical studies had methodological weaknesses and limited relevance to the complexity of human stroke [32]. Using animal models to explore mechanisms is one thing. Relying on them as decisive predictors of therapeutic success is another. And evidence suggests that it remains difficult to predict which animal models will translate successfully to humans [33]. More clinically relevant models do exist in some areas (e.g., breast cancer), but they are often technically more demanding, costly, or less standardized, raising the question of whether convenience sometimes drives model choice [34]. Hence, methods should be aligned with clearly defined research objectives. This includes prioritizing more human-relevant approaches where they can address the research question, including applications in personalized medicine where human-specific variability matters [35].

Where do we go from here? We need cultural and structural change. First, awareness: Training in biomedical science should include core instruction in study design, bias, reproducibility, and the limits of animal models, as well as training in NAMs to enable their appropriate use. Researchers should learn not only how to run experiments, but when not to, for example when a research question can be addressed using established in vitro or computational models without requiring whole-organism validation, and how such principles are implemented in practice.

Second, incentives: Funding agencies and ethics committees can require preregistration of animal studies, robust design features, and adherence to reporting standards such as ARRIVE (Animal Research: Reporting of In Vivo Experiments [36]) and planning standards such as PREPARE (Planning Research and Experimental Procedures on Animals: Recommendations for Excellence [37]) as a condition for funding or approval. Hiring and promotion panels should reward methodological rigor, transparent reporting, and critical justification of animal use, not only publication metrics. Embedding these requirements into funding and ethical review processes would operationalize the principle of “less and better” research [38]. Journal editors and reviewers should also prioritize study quality over positive findings and enforce reporting standards. Unfortunately, adherence to reporting standards such as ARRIVE remains inconsistent in practice [39], suggesting that implementation barriers (potentially informed by behavioural and social science research) need to be better understood. In addition, individual researchers can also act now by preregistering studies and reporting methods transparently, even without formal mandates.

Third, investment in alternatives: Public and private funders should expand targeted programs for the development and validation of human-relevant in vitro and computational approaches, as reflected in recent initiatives such as the NIH’s move to restrict funding for projects relying solely on animal studies [40], and broader policy efforts aimed at replacing animal testing in specific domains [41]. And regulators should define clear pathways for their evaluation and acceptance [42], building on existing initiatives by agencies such as the FDA and EMA that support the evaluation of NAM-based evidence, and existing guidance frameworks for validation and regulatory acceptance [43, 44]. Even clearer regulatory guidance on when non-animal evidence is sufficient would accelerate adoption and reduce unnecessary duplication.

The sequence matters: fewer unnecessary studies, better designed studies, and research aligned with realistic and meaningful objectives.

Animal research has contributed to modern medicine and will likely remain part of it in the coming years. But its legitimacy depends on rigor and relevance. If we use animals only when needed, design studies carefully, and ask questions that truly require them, we honor both science and responsibility. Less, better, and for the right reasons: a standard we should hold ourselves to.

## Data Availability

All data and code that support the findings of this study are available within this manuscript.
